# Ipilimumab-related uveitis and refractory hypotony with a flat chamber in a trabeculectomized eye with exfoliation glaucoma: A case report

**DOI:** 10.1016/j.ajoc.2023.101807

**Published:** 2023-01-20

**Authors:** Naofumi Funagura, Satoshi Fukushima, Toshihiro Inoue

**Affiliations:** a Department of Ophthalmology, Faculty of Life Science, Kumamoto University, 1-1-1, Honjo, Chuo-ku, Kumamoto, Japan; b Department of Dermatology and Plastic Surgery, Faculty of Life Science, Kumamoto University, 1-1-1, Honjo, Chuo-ku, Kumamoto, Japan

**Keywords:** Melanoma, Immune-related adverse events, Uveitis, Hypotony, Exfoliation glaucoma, Trabeculectomy

## Abstract

**Purpose:**

Ipilimumab is an immune checkpoint inhibitor that occasionally causes ophthalmic immune-related adverse events (irAEs) such as dry eye, uveitis, and episcleritis. We report a case of ipilimumab-related uveitis and refractory hypotony with a flat anterior chamber (AC) in a trabeculectomized eye with exfoliation glaucoma.

**Observation:**

A 69-year-old man with a history of cataract surgery and trabeculectomy for exfoliation glaucoma in the right eye presented with blurred vision at 2 months after initiation of ipilimumab for metastatic malignant melanoma (day 0). Although no ophthalmic irAEs were observed at the first visit, he developed iritis, vitreous opacity, and choroidal detachment by day 18.

As a result of the irAEs, the scheduled course of ipilimumab was canceled and he was instead treated with corticosteroids (eye drops and systemic). The symptoms progressed, and on day 32 visual acuity was light perception, with a flat AC, hypotony maculopathy, and severe choroidal detachment in the right eye. The patient received two AC formations with a viscoelastic substance, but the flat AC and hypotony recurred. Because the effects of the surgeries were temporary, high doses of corticosteroids were administered. AC depth, anterior uveitis, intraocular pressure, and choroidal detachment resolved by day 91.

**Conclusions:**

Ophthalmologists and oncologists should be aware of the rare but severe irAEs, and careful follow-up is required for ophthalmic irAEs caused by ipilimumab, especially in cases with a history of glaucoma surgery.

## 1 Introduction

Immune checkpoint inhibitors confer anti-tumor properties to T-cells by binding to the immune checkpoint molecules that they express. Although immune checkpoint inhibitors have clinical benefits, they occasionally cause immune-related adverse events (irAEs).

Ipilimumab, an anti-cytotoxic T-lymphocyte antigen-4 (CTLA-4) monoclonal antibody, is an immune checkpoint inhibitor indicated for the treatment of metastatic melanoma. Ipilimumab inhibits processes involving CTLA-4 and promotes immune responses via T-cell activation, which leads to increased antitumor responses.^1^^,^^2^ However, ipilimumab is associated with several irAEs, with an incidence of 86–93%.^3^^,^^4^ Common systemic irAEs include dermatological, gastrointestinal, hepatic, and endocrine disorders.^3^^,^^4^ Ophthalmic irAEs occur in approximately 1% of patients. Common ophthalmic side effects include dry eye (3–7%), uveitis (<1%), and episcleritis (<1%).^5^

Although there have been reports of uveitis and hypotony induced by immune checkpoint inhibitors,^6^ cases of refractory hypotony with a flat anterior chamber (AC) in a trabeculectomized eye with exfoliation glaucoma have not been reported. Here, we report a case of uveitis and severe hypotony with a flat AC, refractory to surgical treatment and systemic methylprednisolone, after ipilimumab administration.

## 2 Case report

A 69-year-old man presented to the Department of Ophthalmology of our hospital with deteriorating vision in his right eye. He had undergone cataract surgery and trabeculectomy with mitomycin C for exfoliation glaucoma in his right eye at 65 years of age. After trabeculectomy, his right intraocular pressure (IOP) had been controlled at around 10 mmHg. He had also been diagnosed with malignant melanoma in his right big toe and undergone foot surgery to remove the primary lesion and regional lymph nodes 10 months previously. Multiple bone metastases were found, and administration of nivolumab had been started 4 months previously. However, it was not effective, so after 2 months nivolumab was discontinued and he was then treated with ipilimumab; at that time, combination therapy with nivolumab and ipilimumab was not permitted. A drug-induced skin rash occurred after the first course of ipilimumab, and oral prednisolone (20 mg/day) was administered for 5 days and then tapered off.

At the first examination (day 0), his visual acuities were 0 and −0.17 logMAR in the right and left eyes, respectively. The IOPs were 5 and 10 mmHg in the right and left eyes, respectively. He had already received latanoprost eye drops once daily in both eyes for glaucoma. There were no ophthalmic irAEs or other novel findings; the patient was followed-up and a second course of ipilimumab was started.

**Fig. 1:**
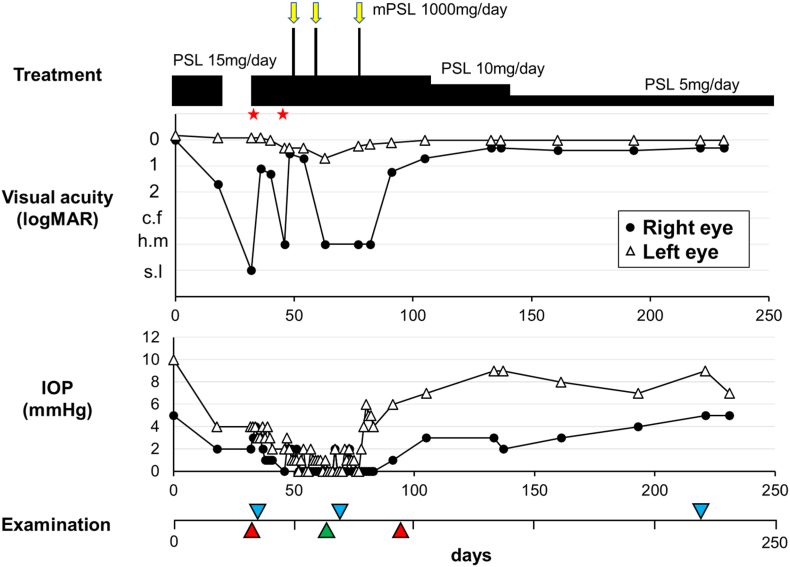
The course of treatment from the date of first examination (day 0). The arrows show the times of steroid pulse therapy (intravenous methylprednisolone [mPSL] 1000 mg/day for 3 days). The star shows the time of anterior chamber formation in the right eye. After two surgeries and three courses of steroid pulse therapy, the visual acuity and intraocular pressure (IOP) gradually recovered. Arrowheads indicate the time of ophthalmic examination [findings are presented in Fig. 2 (green), Fig. 3 (red), and Fig. 4 (blue)]. PSL, prednisolone; c. f, counting finger; h.m, hand motion; s.l, sense light. (For interpretation of the references to colour in this figure legend, the reader is referred to the Web version of this article.)

**Fig. 2:**
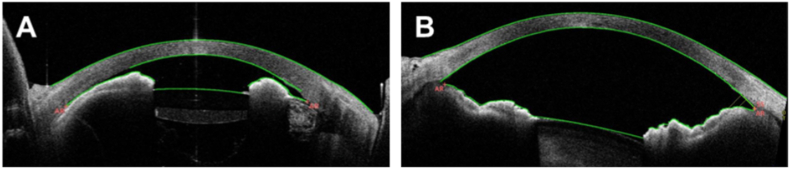
Anterior segment optical coherence tomographic images taken on day 63 after the first examination. A right eye. B left eye. The corneal endothelium and iris almost touch in the right eye. (For interpretation of the references to colour in this figure legend, the reader is referred to the Web version of this article.)

**Fig. 3:**
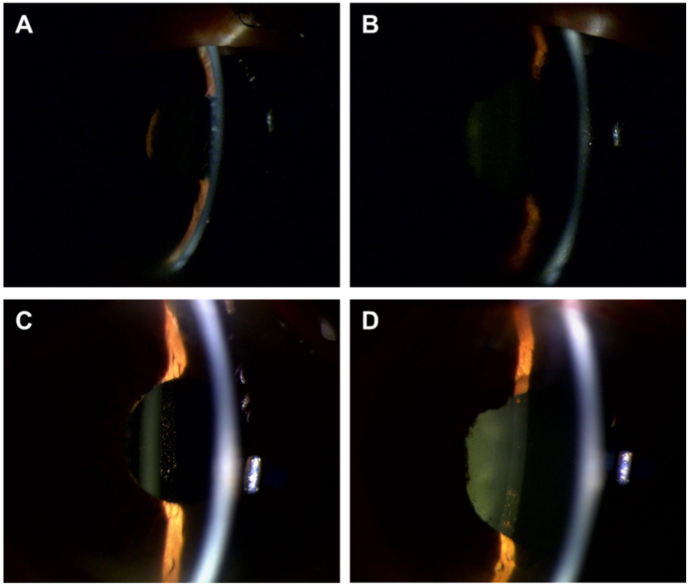
Changes over time in the anterior chamber as revealed by slit lamp examination. The right eye (A) and left eye (B) at 32 days after the first examination. The anterior chamber became very shallow in the right eye. The right eye (C) and left eye (D) at 91 days after the first examination. The anterior chamber depth of the right eye had recovered. The anterior chamber depth of the left eye did not obviously change over the entire period. (For interpretation of the references to colour in this figure legend, the reader is referred to the Web version of this article.)

The patient noticed gradual deterioration of his eyesight. At the second examination (day 18), the visual acuity and IOP in the right eye were 1.7 logMAR and 2 mmHg, respectively. Furthermore, iritis, vitreous opacity, and choroidal detachment developed in the right eye. The scheduled third course of ipilimumab was canceled on suspicion of an irAE, and instead 0.1% betamethasone sodium phosphate eye drops were administered six times daily. The administration of latanoprost eye drops in the right eye was discontinued due to low IOP. On day 32, latanoprost eye drops were discontinued in both eyes and were not administered again until the final visit. The subsequent course of treatment, visual acuity, and IOPs are shown in Fig. 1. In the right eye, on day 32, visual acuity was light perception, with a flat AC, hypotony maculopathy, and severe choroidal detachment. The patient received AC formation by injection of 2.3% sodium hyaluronate solution into the AC and sclerostomy for the choroidal detachment. Postoperatively, the frequency of administration of 0.1% betamethasone sodium phosphate eye drops was reduced to four times daily, and oral prednisolone (15 mg/day) was restarted on day 35. On day 46, after a short period of remission, his visual acuities were hand motion and 0.3 logMAR in the right and left eyes, respectively. The IOPs were 0–2 mmHg in both eyes, and the right AC collapsed again. The patient received AC formation for a second time, which used sub-Tenon's triamcinolone acetonide injection in addition to injection of 2.3% sodium hyaluronate solution in the right eye on the same day. Although ophthalmic findings improved to some extent after the surgery, the IOPs remained at 0–2 mmHg in both eyes. Therefore, steroid pulse treatment was started on day 50 (1000 mg intravenous methylprednisolone). The right AC started to become shallow on day 55 and had almost collapsed by day 63 (Fig. 2). Steroid pulse treatment had been performed three times by day 78. AC depth, anterior uveitis, IOPs, and choroidal detachment recovered by day 91 (Fig. 3). On day 105, visual acuities were 1.7 and 0 logMAR, and IOPs were 3 and 7 mmHg in the right and left eyes, respectively. The filtering bleb in the right eye was almost flat and its appearance did not change throughout the treatment period.

**Fig. 4:**
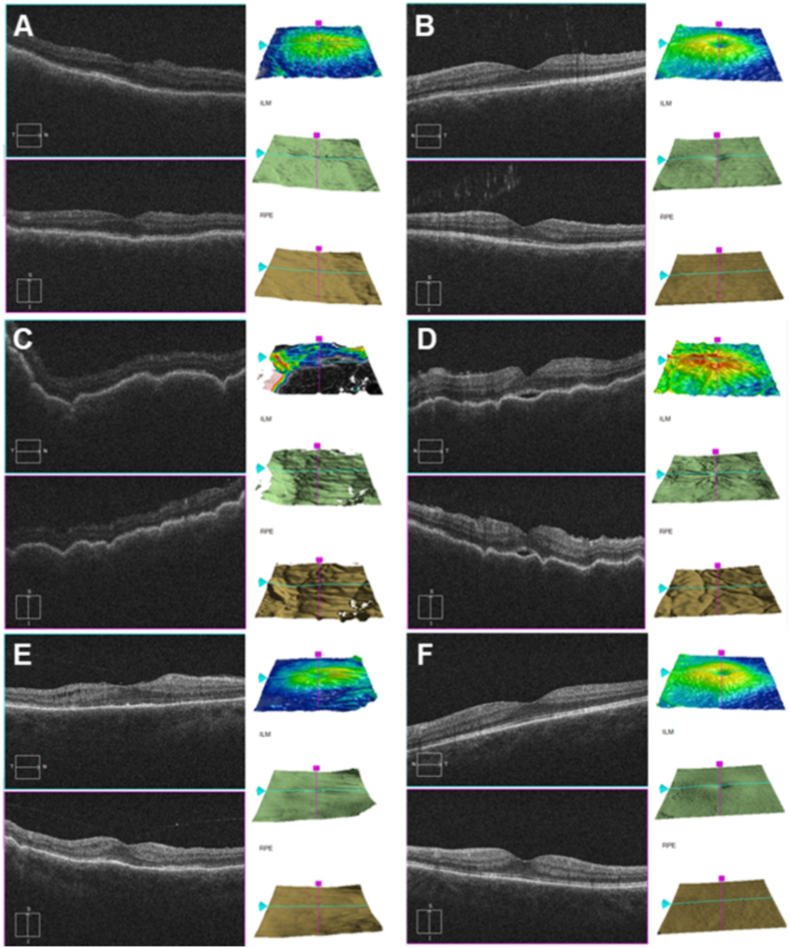
Changes in hypotonic maculopathy over time as revealed by optical coherence tomography. A, B: Images taken on day 36 after the first examination of the right eye (A) and left eye (B); the right eye exhibited hypotonic maculopathy and serous retinal detachment. C, D: Images taken on day 70 [right (C) and left (D) eyes]; maculopathy was evident in both eyes. E, F: Images taken on day 221 [right (E) and left (F) eyes]; maculopathy had improved in both eyes, although a slight retinal fold and subretinal fluid were present in the right eye. (For interpretation of the references to colour in this figure legend, the reader is referred to the Web version of this article.)

The sizes of the lymph nodes and subcutaneous metastases on contrast-enhanced computed tomography (CT) were reduced, while the liver and bone metastases remained stable. The dose of oral prednisolone was gradually decreased to 5 mg/day by day 133. However, brain metastases were detected on brain CT on day 193. After discussion with the attending dermatologist, radiation therapy for brain metastasis was initiated on day 210. Pembrolizumab was administered from day 238. A drug-induced skin rash occurred after the second administration of pembrolizumab, so the dose of oral prednisolone was increased to 15 mg/day from day 277. On day 364, brain magnetic resonance imaging (MRI) was performed because the patient complained of dizziness, which showed that the metastasis had disseminated to the meninges. At the final visit to the Department of Ophthalmology (day 378), the visual acuity and IOP in his right eye were 0.1–0.2 logMAR and 11 mmHg, respectively. The reason for the incomplete recovery of visual acuity was probably prolonged hypotony maculopathy (Fig. 4). We continued 0.1% betamethasone sodium phosphate eye drops four times daily until the final visit. Eventually, symptoms of brain metastasis (spasms and insomnia) led to discontinuation of pembrolizumab. The patient was transferred to another hospital for palliative care and died on day 432.

## 3 Discussion

In ophthalmologic irAEs, uveitis is typically bilateral and affects the anterior segment of the eye.^6^^,^^7^ Uveitis caused by ipilimumab occurs as a result of an excessive immune response to melanocytes. Malignant melanoma is derived from melanocytes, so antigen recognition by ipilimumab-activated T-cells can occur in a uveal tract containing melanocytes and trigger uveitis. Hypotony also occurred in both eyes in this patient, and the right AC almost collapsed. Previous reports have shown that immune checkpoint inhibitors occasionally cause uveitis and hypotony.^8^, ^9^, ^10^ It is thought that hypotony is caused by uveitis, trauma, medications, and ocular ischemia.^11^ Our patient exhibited ipilimumab-related uveitis in both eyes, which probably led to hypotony. Uveitis can cause ciliary dysfunction, which can in turn decrease aqueous humor secretion and increase uveoscleral outflow.^12^ This was the first case of immune checkpoint inhibitor-related ocular hypotony with a flat AC in a trabeculectomized eye.

Considering the difference in the severity of side effects between the right and left eyes, the flat AC in the right eye could have been related to exfoliation glaucoma. Exfoliation syndrome is associated with blood-aqueous barrier dysfunction,^13^^,^^14^ so trabecular outflow may have been impaired in the right eye. On the other hand, there was no obvious increase in the filtration of anterior aqueous humor associated with trabeculectomy, because the filtration bleb in the right eye remained flat after the first ophthalmological examination. Based on the flattened filtration bleb, over-filtration of aqueous humor might not have greatly influenced the hypotony and flat AC. Because we did not measure the change in aqueous humor outflow, we cannot not entirely rule out the possible influence of trabeculectomy on the difference in response between the right and left eyes. In this case, hypotony caused the maculopathy and vision loss. Therefore, when uveitis-induced hypotony occurs, control of inflammation may be key to maintain visual function. It should be noted that systemic steroids often have multiple side effects, such as susceptibility to infections, diabetes, and whirlbone necrosis. However, in a previous report, systemic steroid therapy did not affect the overall survival or time to treatment failure in cases with irAEs.^15^ Therefore, it is unclear whether the multiple steroid pulse treatments were effective for the ophthalmic irAEs seen in this case. It is important to establish standard treatments for cases with irAEs with severe hypotony.

## 4 Conclusion

Although ipilimumab occasionally causes several ophthalmic irAEs, severe ophthalmic side effects are rare. Ophthalmologists and oncologists should be aware of the rare but severe irAEs, and careful follow-up may be required in cases with ipilimumab administration, especially for those with a history of glaucoma surgery.

## Patient consent

Written informed consent was obtained from the patient's family for publication of this case report and the accompanying images.

## Funding

No funding or grant support.

## Authorship

All authors attest that they meet the current ICMJE criteria for authorship.

## Declaration of competing interest

The authors declare that they have no known competing financial interests or personal relationships that could have appeared to influence the work reported in this paper.
